# PAX8‐positive conventional urothelial carcinomas of the urinary bladder and their distinct molecular profiles – A clinicopathologic study of 101 consecutive cases with next‐generation sequencing in 20 cases

**DOI:** 10.1111/his.70112

**Published:** 2026-02-06

**Authors:** Sarah Mae Lammert, Wendy Luo, Allen C Zhu, Peng Wang, Tatjana Antic, Jung Woo Kwon

**Affiliations:** ^1^ Department of Pathology The University of Chicago Chicago Illinois USA; ^2^ Pritzker School of Medicine The University of Chicago Chicago Illinois USA

**Keywords:** bladder, PAX8, *TERT* promoter, *TSC1*, urothelial carcinoma

## Abstract

**Aims:**

PAX8 immunohistochemistry (IHC) is often used to distinguish urothelial carcinomas (UCs) from tumours of renal and Mullerian origin. However, some UCs have been shown to be PAX8‐positive. This study investigates the frequency of PAX8‐positive conventional UCs of the urinary bladder without subtype morphology and/or divergent differentiation and their molecular profiles.

**Methods:**

One hundred and one consecutive transurethral resections of the urinary bladder from 2019 to 2022 with a diagnosis of conventional urothelial carcinoma (UC) without subtype morphology and/or divergent differentiation were retrospectively reviewed. Representative sections were selected for whole‐slide PAX8 IHC (10336‐1‐AP; polyclonal). Next‐generation sequencing (NGS) was performed on all PAX8‐positive cases and on a subset of PAX8‐negative cases.

**Results:**

PAX8 IHC was positive in 10% (10/101) of cases. Twenty cases underwent NGS, including all 10 PAX8‐positive UCs. All PAX8‐positive UCs had *TERT* promoter mutations. *TSC1* alterations, *NOTCH1* loss and *WT1* loss were more frequent in the PAX8‐positive cases compared with the PAX8‐negative cases. *RB1* loss was not seen in the PAX8‐positive UCs, while it was present in 40% of the PAX8‐negative UCs.

**Conclusions:**

A subset of conventional UCs of the urinary bladder without subtype morphology and/or divergent differentiation are PAX8‐positive and have frequent alterations in *TERT* promoter, *TSC1*, *NOTCH1*, and *WT1*, with an absence of *RB1* loss. PAX8 positivity should be interpreted with caution if UC is in the differential, and NGS could be helpful in diagnostic workups as this study shows PAX8‐positive UCs may have a distinct molecular profile.

## Graphical abstract

PAX8 expression in urothelial carcinoma was investigated in 101 consecutive transurethral resections of the urinary bladder. PAX8 was positive in 10% of cases, with targeted molecular testing of these cases revealing universal *TERT* promoter mutations and frequent alterations in *TSC1*, *NOTCH1* and *WT1*.

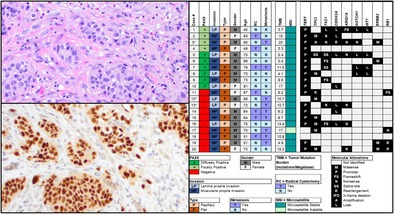

**Abbreviations**
CNV: Copy number variant
DNA: Deoxyribonucleic acid
EGFR: epidermal growth factor receptor
FC: fold change
*FGFR3*
: fibroblast growth factor receptor 3
IHC: immunohistochemistry
MP: muscularis propria
mRNA: messenger ribonucleic acid
mTOR: mammalian target of rapamycin
NGS: next‐generation sequencing
*NOTCH1*
: neurogenic locus notch homologue protein 1
*PAX*
: paired box
*PAX5*
: paired box 5
*PAX6*
: paired box 6
*RB1*
: RB transcriptional corepressor 1
*TERT*
: telomerase reverse transcriptase
TMB: tumour mutational burden
*TP53*
: tumour protein 53
*TSC1*
: tuberous sclerosis complex 1
TURBTs: transurethral resections of the urinary bladder
UC: urothelial carcinoma
UTUC: upper tract urothelial carcinoma
WHO: World Health Organization
*WT1*
: Wilms' tumour gene

## Introduction

Invasive urothelial carcinoma (UC) is the most common form of bladder cancer. Invasive UC can exhibit a wide variety of morphology and is notable for its diverse histological subtypes and divergent differentiations, with 14 histologic subtypes and 3 divergent differentiations described in the most recent World Health Organization (WHO) Classification of Tumours.^1^, ^2^, ^3^, ^4^, ^5^ Beyond the WHO Classification of Tumours, urothelial carcinomas (UCs) with other unusual morphology also have been described, such as UCs with chordoid and myxoid features.^6^ As such, when one encounters a carcinoma with unusual morphology in the urinary bladder, a diagnosis based on morphology alone can be challenging, especially when the patient has a history of other cancers. Immunohistochemistry (IHC) can be helpful in such settings, particularly GATA3 and p63, which are usually positive in UC, although their positivity varies between subtypes and can be lost in poorly differentiated or metastatic tumours.^3^, ^7^

*PAX8* is a member of the paired box (*PAX*) gene family located on chromosome 2q14 which mainly encodes transcription factors involved in the development of the thyroid, Mullerian system, kidney and upper urinary tract tissue.^8^, ^9^, ^10^, ^11^, ^12^, ^13^, ^14^, ^15^, ^16^, ^17^, ^18^, ^19^, ^20^, ^21^ Within the realm of diagnostic surgical pathology, PAX8 IHC is a useful lineage stain that is positive in neoplasms of the thyroid, Mullerian organs and kidney.^9^, ^11^, ^15^, ^17^, ^18^, ^19^, ^21^ In the genitourinary system, PAX8 staining has historically been used to differentiate UC from tumours of these aforementioned origins, especially nephrogenic adenomas.^11^, ^12^, ^15^, ^17^, ^18^, ^19^, ^21^, ^22^, ^23^, ^24^

Despite this, PAX8 staining in UC has been variably described and may be a diagnostic pitfall if relied upon to narrow down a differential. Multiple case series have described PAX8 staining in nested variants of UC and in those from upper tract locations.^8^, ^11^, ^12^, ^16^, ^19^, ^24^ Few others have shown PAX8 positivity in non‐invasive urothelial neoplasms of the urinary bladder as well as in other morphological subtypes of invasive UC (i.e. sarcomatoid).^8^, ^9^, ^12^, ^16^, ^25^, ^26^, ^27^, ^28^ Although cross‐reactivity with expression of paired box 5 (*PAX5*) and/or paired box 6 (*PAX6*) may occur with both monoclonal and polyclonal *PAX8* antibodies used for IHC, a recent study by Gorbokon *et al*.^9^ demonstrated PAX8 (monoclonal) staining in subsets of UC after a meticulous validation of their assays to minimize antibody‐specific cross‐reactivities. Additionally, studies by Pellizzari *et al*.^16^ and Xiang *et al*.^21^ showed that positive staining of urothelial neoplasms with a polyclonal PAX8 stain corresponded with the presence of *PAX8* messenger ribonucleic acid (mRNA) isoforms.

As previously stated, PAX8 positivity has been noted in UC with nested morphology and is variably seen in other morphological subtypes as well as in noninvasive urothelial neoplasms.^8^, ^9^, ^14^ However, relatively little data exist evaluating PAX8 staining in conventional UC of the urinary bladder without subtype morphology. Therefore, this study aims to evaluate PAX8 IHC in conventional UCs of the urinary bladder and their underlying molecular profiles.

## Materials and Methods

One hundred and one consecutive transurethral resections of the urinary bladder (TURBTs) from 2019 to 2022 with a diagnosis of conventional UC without subtype morphology and/or divergent differentiation as defined by the WHO^2^ were reviewed and categorized as either flat‐type or papillary. Invasion was defined by tumour presence in the lamina propria (LP) or in the muscularis propria (MP). Clinicopathologic parameters were collected from the electronic medical record. Informed consent was waived by the University of Chicago Institutional Review Board (IRB24‐2079).

Cases were reviewed and a representative tumour‐rich paraffin‐embedded block was selected for whole‐slide PAX8 IHC (10336‐1‐AP; polyclonal). PAX8 staining distribution was described as diffuse if ≥50% of tumour cell nuclei were positive or as focal if <50% of tumour cell nuclei were positive. Staining intensity was initially assigned as none, weak or strong. On independent review by two pathologists, the distinction of weak staining intensity versus no staining was not considered reproducible. Therefore, only cases deemed to have strong PAX8 staining were counted as positive.

Next‐generation sequencing (NGS) was performed on all PAX8‐positive cases with microdissection of the PAX8‐positive tumour cells. A subset of PAX8‐negative cases also underwent NGS as a control. A representative formalin‐fixed, paraffin‐embedded block was selected for NGS within the verified University of Chicago Medicine OncoPlus (UCM‐OncoPlus) panel, which is a hybrid‐capture panel targeting 1213 cancer‐associated genes. Deoxyribonucleic acid (DNA) extraction, DNA quantification, library preparation and NGS were performed as described in the validation study by Kadri *et al*.^29^ Analysis was performed using a high‐performance computing system (Center for Research Informatics, University of Chicago) and an in‐house developed bioinformatics pipeline. Copy number variant (CNV) Kit14 software with additional in‐house intra‐run normalization with comparison to a pooled cohort of clinical controls was used to calculate copy number results. The threshold for arm‐level copy number changes was an arm average fold change (FC) <0.75 designated as deleted and an arm average FC >1.5 designated as amplified. Somatic variant calls were inspected using Integrated Genomics Viewer (Broad Institute, MIT Harvard, Cambridge, MA). Cosmic^30^ and ClinVar^31^ were used as additional tools to analyse and confirm detected variants.

RNA‐seq was performed with the University of Chicago's RNA Oncoplus Assay for gene fusion analysis. In addition to whole exon coverage of 1213 cancer‐associated genes, the panel is capable of detecting known and novel fusions involving any of the 1213 genes. RNA was isolated using the FFPE RNA Extraction Kit (Qiagen, Hilden, Germany). The RNA was subjected to library preparation using adapter molecules containing patient‐specific index sequences (KAPA Stranded RNA‐Seq Kit with RiboErase; Kapa Biosystems, Wilmington, MA). The libraries were sequenced in a rapid run mode on a NovaSeq 6000 system (Illumina, San Diego, CA). The sequencing data were analysed for fusions via a custom‐designed bioinformatics pipeline on a University of Chicago HIPAA‐compliant high‐performance computing system, using the hg19 (GRCh37) human genome reference sequence for alignment.^29^

## Results

**Figure 1:**
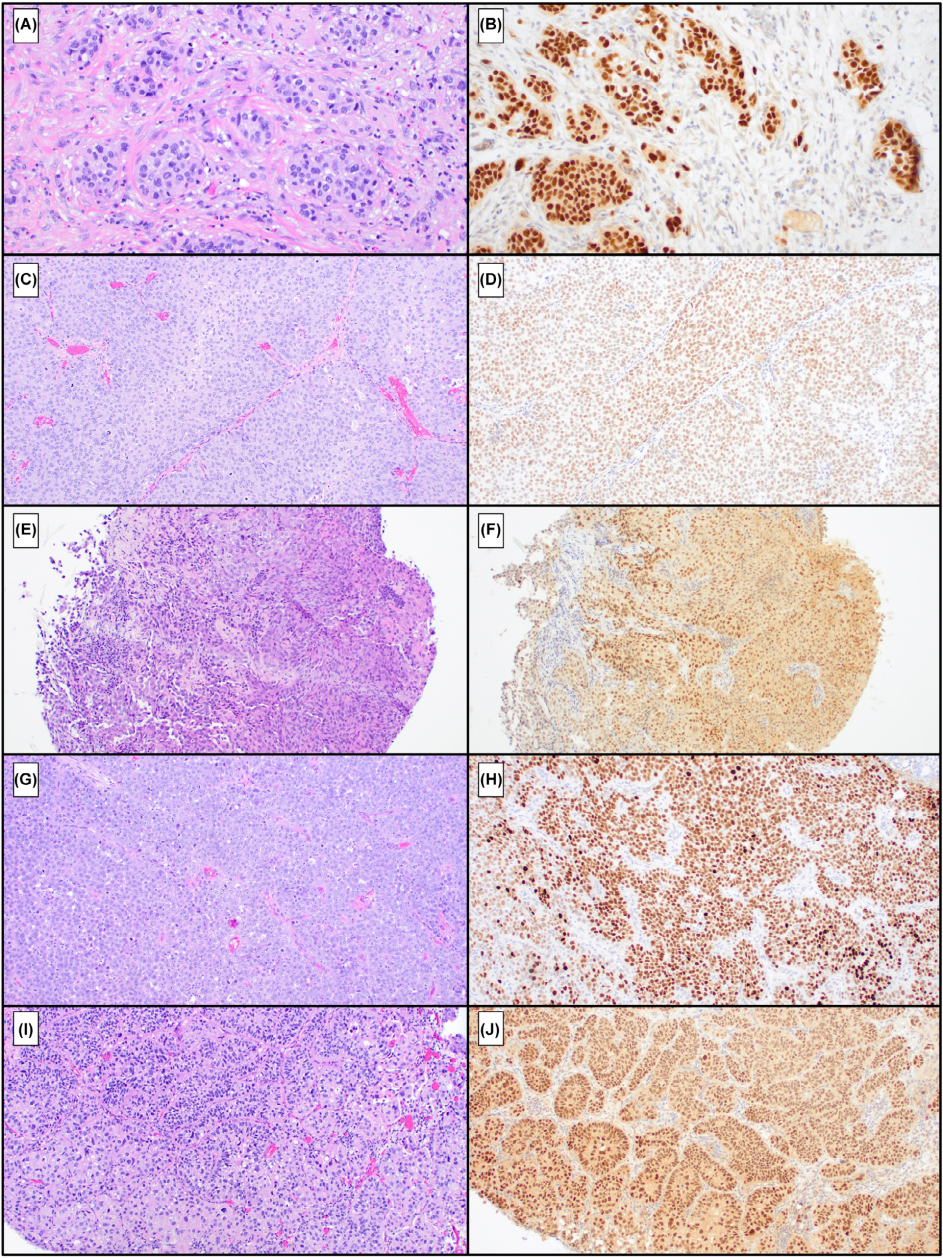
Representative haematoxylin and eosin stains (**A, C, E, G, I**) with corresponding PAX8 immunostains (**B, D, F, H, J**) from representative PAX8‐positive cases.

Clinicopathologic parameter**s** are detailed in Table 1. Tumour cells stained for PAX8 in 10% (*N* = 10/101) of cases (Figure 1). Six (60% of PAX8‐positive cases, 6% of total) of these exhibited diffuse staining and 4 (40% of PAX8‐positive cases, 4% of total) showed focal staining. One case with diffuse PAX8 staining was evaluated with GATA‐3 and CK7, both of which showed diffuse positivity in the surface and invasive UC components; the remaining cases were not stained, given their conventional morphology. Morphologically, 6 (60%) of the PAX8‐positive cases were papillary and 4 (40%) had flat‐type architecture. Additionally, 3 (30%) of the PAX8‐positive cases had metastatic lesions. The average age in these PAX8‐positive cases was 70 (range = 40–89), with a male predominance (*N* = 7; 70%). PAX8‐positive case clinicopathologic parameters are summarized in Table 2.

**Table 1:** Clinicopathologic parameters of all cases

Characteristics	All cases (*N* = 101)
Median age (years)	75
Sex	
Male, *N* (%)	66 (65%)
Female, *N* (%)	35 (35%)
Type	
Flat, *N* (%)	51 (50%)
Papillary, *N* (%)	50 (50%)
Extent of Invasion	
Lamina propria invasion only, *N* (%)	35 (35%)
Muscularis propria invasion, *N* (%)	66 (65%)
Distant metastasis, *N* (%)	31 (31%)
PAX8 Immunohistochemistry	
Positive, *N* (%)	10 (10%)
Diffuse (≥50%), *N*	6
Focal (<50%), *N*	4
Negative, *N* (%)	91 (90%)

**Table 2:** Clinicopathologic parameters of sequenced cases

Characteristics	PAX8‐positive cases (*N* = 10)	Sequenced PAX8‐negative cases (*N* = 10)
Median age (years)	70	68
Sex		
Male, *N* (%)	7 (70%)	7 (70%)
Female, *N* (%)	3 (30%)	3 (30%)
Type		
Flat, *N* (%)	4 (40%)	6 (60%)
Papillary, *N* (%)	6 (60%)	4 (40%)
Extent of invasion		
Lamina propria invasion, *N* (%)	4 (40%)	3 (30%)
Muscularis propria invasion, *N* (%)	6 (60%)	7 (70%)
Distant metastasis, *N* (%)	3 (30%)	7 (70%)
History of upper tract urothelial carcinoma, *N* (%)		
Total	1 (1%)	2 (2%)
Diffuse PAX8 staining (≥50%), *N*	0	
Focal PAX8 staining (<50%), *N*	1	
Median [range] follow‐up time (months)		
	42 [15–74]	30 [15–56]

**Figure 2:**
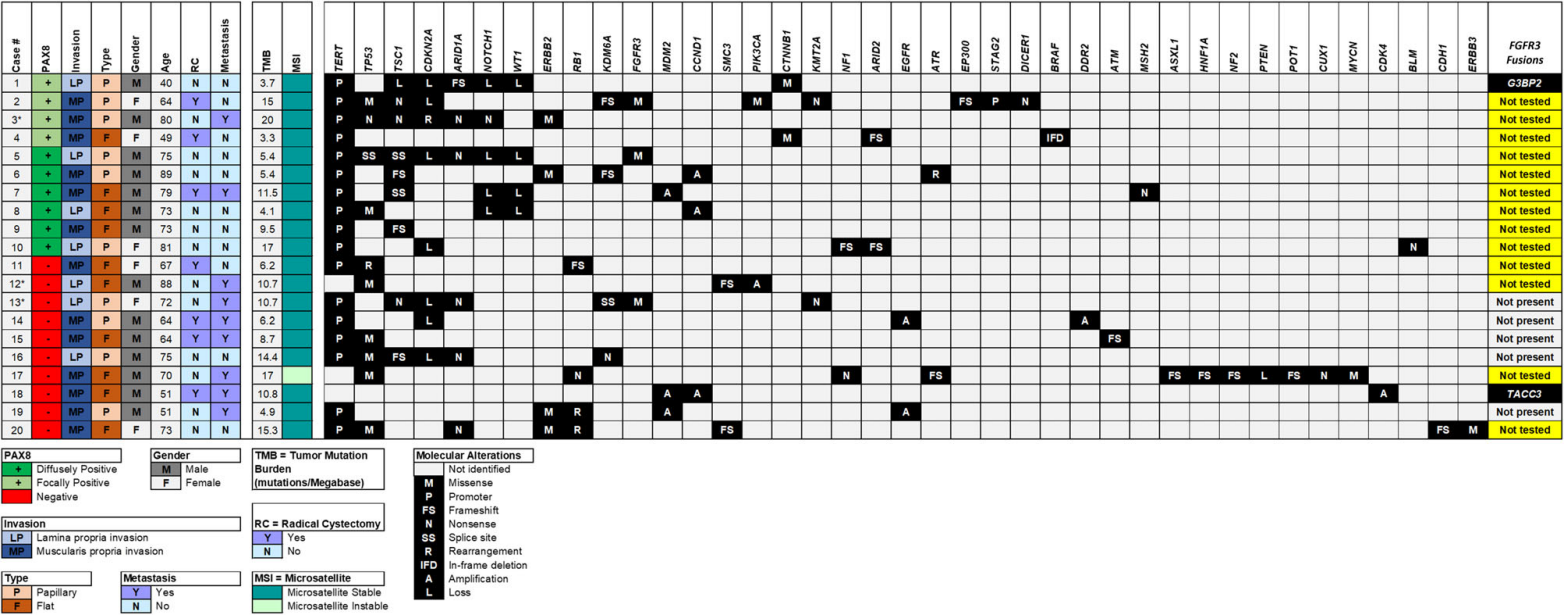
Molecular profile of sequenced PAX8‐positive and PAX8‐negative cases. *Patients with a history of upper tract urothelial carcinoma (UTUC) before or concurrent with invasive urothelial carcinoma (UC) of the urinary bladder: non‐asterisk cases had no history of UTUC.

A total of 20 cases underwent NGS, including the 10 PAX8‐positive cases and 10 PAX8‐negative cases. RNA fusion testing was performed in 7 of these sequenced cases. One PAX8‐positive case and one PAX8‐negative case demonstrated *FGFR3* fusions with *G3BP2* and *TACC3*, respectively. An additional 3 cases (2 PAX8‐positive and 1 PAX8‐negative) had missense mutations in *FGFR3*. NGS and RNA fusion testing results are illustrated in Figure 2. Of these sequenced cases, 1 of the focally PAX8‐positive cases had a history of upper tract urothelial carcinoma (UTUC) preceding TURBT status‐post neoadjuvant chemotherapy and nephroureterectomy. Additionally, 2 of the PAX8‐negative cases had a history of UTUC. None of the remaining sequenced PAX8‐positive and negative cases had a history of UTUC or development of UTUC during the follow‐up period noted in Table 2. All 10 PAX8‐positive cases had telomerase reverse transcriptase (*TERT*) promoter mutations, compared with 7/10 of the sequenced PAX8‐negative cases. Several additional aberrations were more frequently found in the PAX8‐positive cases compared with the PAX8‐negative cases. These included tuberous sclerosis complex 1 (*TSC1*) alterations (70% vs. 20%), neurogenic locus notch homologue protein 1 (*NOTCH1*) loss (50% vs. 0%) and Wilms' tumour gene (*WT1*) loss (40% vs. 0%). RB transcriptional corepressor 1 (*RB1*) loss was not seen in the PAX8‐positive cases, while it was seen in 40% of the PAX8‐negative cases. CNV analysis did not show significant recurrent copy number changes and there were no notable differences in tumour mutational burden (TMB) between the groups. Clinicopathologic parameters of the sequenced groups did not differ greatly (Table 2).

## Discussion

Among the cases reviewed, this study demonstrates PAX8 staining in 10% of conventional UCs of the urinary bladder. This is the first large study to investigate PAX8 IHC expression in conventional UC of the urinary bladder with molecular testing. The findings in this study may suggest PAX8‐positive UCs have a distinct molecular profile with frequent alterations in *TERT* promoter, *TSC1*, *NOTCH1* and *WT1*, with an absence of *RB1* loss. Our findings lead us to two main discussion points: the circumstances and utility of PAX8 staining in UC and the presence of specific molecular aberrations seen in our subset of PAX8‐positive cases.

As aforementioned, PAX8 staining has been reported in UTUC, a few noninvasive bladder tumours, and certain morphological subtypes of invasive urinary bladder UC.^8^, ^9^, ^11^, ^12^, ^16^, ^19^, ^24^, ^25^, ^26^, ^27^, ^28^ While one of our focally PAX8‐positive cases had a history of UTUC, the remaining 9 cases had no history of UTUC or evidence of progressive disease involving the upper urinary tracts (Table 2). Additionally, the molecular profiles of the cases with an UTUC history did not share overlapping genetic aberrations that distinguished them from the remainder of the sequenced cohort. Multiple studies^8^, ^9^, ^11^, ^12^, ^16^, ^19^, ^24^, ^25^, ^26^, ^27^, ^28^ have emphasized that PAX8 IHC must be cautiously interpreted when UC is in the differential. Our findings provide further evidence that PAX8 staining can also be seen in conventional UCs of the urinary bladder. Being cognizant of this will help prevent premature exclusion of UC based on PAX8 staining, especially in poorly differentiated neoplasms, metastatic lesions, or where nephrogenic adenoma and similar neoplasms are in the differential.

PAX8 staining may reflect the presence of nuclear *PAX8* transcripts or, due to cross‐reactivity, other *PAX* proteins. While our study did not assess *PAX8* mRNA in tumours with positive PAX8 IHC, other studies have shown a correlation.^16^, ^21^ Pellizzari *et al*.^16^ found *PAX8* mRNA isoforms in the developing human bladder but not in normal bladder mucosa. In noninvasive bladder tumours, *PAX8* mRNA was present, aligning with positive PAX8 staining (polyclonal). This suggests *PAX8* expression in urothelial tumours may be linked to dedifferentiation and reactivation of embryonal transcription pathways.^16^ More recently, Xiang *et al*.^21^ observed *PAX8* mRNA isoforms in UTUC that also showed positive PAX8 staining (polyclonal).

The overexpression or upregulation of *PAX* genes can lead to increased or dysregulated transcription and subsequent cell proliferation with oncogenesis. *PAX8* has been implicated directly and indirectly in the oncogenesis of multiple tumours.^8^, ^10^, ^12^, ^14^, ^16^, ^21^, ^32^, ^33^, ^34^, ^35^, ^36^, ^37^ Most research has focused on *PAX8*'s role in the pathogenesis of traditionally PAX8‐positive tumours. As such, it is unclear whether the positive PAX8 staining in UC indicates a connection to *PAX8* gene‐related functions that may contribute to tumour development. Our findings do not suggest that PAX8 IHC should be utilized in the routine workup of UC, as it is positive in only a minority of urothelial tumours. It remains to be seen if there might be some utility for *PAX8* detection through molecular analysis of urine in patients with a history of PAX8‐positive UC.^21^

Over the past decade, NGS has transformed the characterization of neoplasms by revealing their molecular profiles and identifying potential therapeutic targets. Over 50 significantly mutated genes have been identified in UC, including tumour protein 53 (*TP53*), cyclin‐dependent kinase inhibitor 2A (*CDKN2A*), fibroblast growth factor receptor 3 (*FGFR3*), phosphatidylinositol‐4,5‐bisphosphate 3‐kinase catalytic subunit alpha (*PIK3CA*), *TERT* and *RB1*, to name a few. Classification of UC in regard to molecular aberrations has been described in several forms, including by mutational signature clusters,^38^, ^39^ molecular taxonomy,^38^, ^39^, ^40^, ^41^ molecular profiles with associated prognostic outcomes and by molecular profiles of specific variants.^38^, ^39^ Within our sequenced cases, PAX8‐positive UCs showed more frequent alterations in *TERT* promoter, *TSC1*, *NOTCH1* and *WT1*, with an absence of *RB1* loss compared with PAX8‐negative UCs.

The *TERT* gene encodes a component of the enzyme telomerase and in normal tissues is silenced, leading to telomere shortening with successive cell divisions.^42^ *TERT* promoter mutations are frequently seen in bladder UCs and are often associated with a poor prognosis.^2^, ^43^, ^44^, ^45^, ^46^, ^47^, ^48^, ^49^, ^50^ Their presence in PAX8‐positive metastatic or poorly differentiated tumours may suggest UC, especially since all our PAX8‐positive cases harboured *TERT* promoter mutations. In contrast, *TERT* promoter mutations are uncommon in other PAX8‐positive tumours, reported in approximately 2% of renal, 5% of uterine and 7% of fallopian tube/ovarian tumours.^43^, ^44^, ^45^

*TSC1*, a tumour suppressor gene on chromosome 9q34, is mutated in approximately 9% of bladder UCs.^43^, ^44^, ^45^, ^51^, ^52^, ^53^ In contrast, *TSC1* mutations were found in 70% of our PAX8‐positive cases. These mutations are less common in other PAX8‐positive tumours, reported in approximately 5% of renal, 4% of uterine and 1% fallopian tube/ovarian tumours.^43^, ^44^, ^45^ Hence, co‐occurrence of *TERT* promoter and *TSC1* mutations in a poorly differentiated PAX8‐positive tumour may support a diagnosis of UC. *TSC1* mutations may affect therapy response, sensitizing tumours to mammalian target of rapamycin (mTOR) inhibitors, epidermal growth factor receptor (EGFR) inhibitors and heat shock protein 90 (HSP90) inhibitors.^51^, ^53^, ^54^

*NOTCH1*, a key regulator of development and tissue repair, is mutated in approximately 5% of bladder UCs.^43^, ^44^, ^45^, ^55^ We found *NOTCH1* mutations in 50% of PAX8‐positive cases. Such mutations are less common in other PAX8‐positive tumours, reported in approximately 1% of renal, 8% of uterine and 2% of fallopian tube/ovarian tumours.^43^, ^44^, ^45^ Diagnostically, the presence of both *TERT* promoter and *NOTCH1* mutations in a poorly differentiated PAX8‐positive malignancy may suggest a diagnosis of UC. In UC, loss of *NOTCH* signalling may promote tumourigenesis by disrupting epithelial stability, and low *NOTCH1* expression is linked to worse survival in certain UC subtypes.^56^, ^57^, ^58^

*WT1*, a transcription factor on chromosome 11p13 essential for genitourinary development, is altered in approximately 3% of bladder UCs.^43^, ^44^, ^45^, ^59^, ^60^ We found *WT1* alterations in 40% of PAX8‐positive cases and in none of the sequenced PAX8‐negative cases. These mutations are rare in other PAX8‐positive tumours, reported in approximately <1% of renal, 2% of uterine and 3% fallopian tube/ovarian tumours.^43^, ^44^, ^45^ *WT1* can act as a tumour suppressor or oncogene, and in UC, its loss may disrupt epithelial–mesenchymal transitions, destabilizing urothelial differentiation and promoting proliferation.^43^, ^44^, ^45^, ^60^

This study was conducted at a single institution and could benefit from a larger number of cases. In terms of the PAX8 IHC stain used, cross‐reactivity with *PAX5* and *PAX6* expression has been described when using a polyclonal antibody.^61^, ^62^ However, multiple studies have shown concurrent positive polyclonal PAX8 staining with the presence of *PAX8* mRNA in urothelial neoplasms.^9^, ^16^, ^21^ Additionally, multiple studies that have investigated PAX8 IHC expression in UC have used polyclonal antibodies.^12^, ^18^, ^22^, ^26^ Regardless, the reported 10% PAX8 positivity rate is specific to the antibody clone used at our institution, and the interpretive criteria used in this assay may not directly translate to monoclonal PAX8 assays performed in other laboratories. Ideally, future studies would reproduce these findings using monoclonal PAX8 IHC. Additionally, a direct biochemical connection between the gene mutations seen in our PAX8‐positive cases and the expression of PAX8 IHC is not established and is an area to expound upon in future research on this topic.

## Conclusion

This study highlights the presence of PAX8 staining in a subset of conventional UCs of the urinary bladder without subtype morphology and/or divergent differentiation, providing new insights into PAX8 IHC patterns. The observed concurrence between PAX8 positivity and specific molecular alterations, including *TERT* promoter, *TSC1*, *NOTCH1* and *WT1*, along with an absence of *RB1* loss, may suggest that PAX8‐positive UCs represent a distinct molecular subgroup. Although our findings do not support PAX8 staining in routine UC diagnostics, PAX8 positivity seen during workup of a complex differential, especially in cases of poorly differentiated or metastatic carcinomas, should not exclude UC. Additionally, PAX8 positivity in UC may suggest the presence of the associated molecular aberrations found in our study that have therapeutic implications.

## Author contributions

Tatjana Antic, and Jung Woo Kwon, contributed to the study conception and design. Material preparation, data collection and analysis were performed by all authors: Sarah Mae Lammert, Wendy Luo, Allen C. Zhu, Peng Wang, Tatjana Antic. The first draft of the manuscript was written by Sarah Mae Lammert, and Jung Woo Kwon and all authors commented on previous versions of the manuscript. All authors read and approved the final manuscript.

## Conflict of interest statement

All authors have nothing to disclose.

## Informed consent

Waived by the University of Chicago Institutional Review Board (IRB24‐2079).

## Data Availability

The data that support the findings of this study are available from the corresponding author upon reasonable request.
